# The biological properties of different Epstein-Barr virus strains explain their association with various types of cancers

**DOI:** 10.18632/oncotarget.14380

**Published:** 2016-12-30

**Authors:** Ming-Han Tsai, Xiaochen Lin, Anatoliy Shumilov, Katharina Bernhardt, Regina Feederle, Remy Poirey, Annette Kopp-Schneider, Bruno Pereira, Raquel Almeida, Henri-Jacques Delecluse

**Affiliations:** ^1^ German Cancer Research Centre (DKFZ), Unit F100, 69120 Heidelberg, Germany; ^2^ Inserm unit U1074, DKFZ, 69120 Heidelberg, Germany; ^3^ Institute for Diabetes and Obesitas, Monoclonal Antibody Core Facility, German Research Center for Environmental Health, Helmholtz Zentrum München, 81377 Munich, Germany; ^4^ German Cancer Research Centre (DKFZ), Unit C060, 69120 Heidelberg, Germany; ^5^ Differentiation and Cancer Group, IPATIMUP, Rua Dr Roberto Frias s/n, 4200 - 465 Porto, Portugal

**Keywords:** human tumor viruses, Epstein-Barr virus strains, viral infection and transformation, host-virus interactions, carcinoma and lymphoma

## Abstract

The Epstein-Barr virus (EBV) is etiologically associated with the development of multiple types of tumors, but it is unclear whether this diversity is due to infection with different EBV strains. We report a comparative characterization of SNU719, GP202, and YCCEL1, three EBV strains that were isolated from gastric carcinomas, M81, a virus isolated in a nasopharyngeal carcinoma and several well-characterized laboratory type A strains. We found that B95-8, Akata and GP202 induced cell growth more efficiently than YCCEL1, SNU719 and M81 and this correlated positively with the expression levels of the viral BHRF1 miRNAs. In infected B cells, all strains except Akata and B95-8 induced lytic replication, a risk factor for carcinoma development, although less efficiently than M81. The panel of viruses induced tumors in immunocompromised mice with variable speed and efficacy that did not strictly mirror their *in vitro* characteristics, suggesting that additional parameters play an important role. We found that YCCEL1 and M81 infected primary epithelial cells, gastric carcinoma cells and gastric spheroids more efficiently than Akata or B95-8. Reciprocally, Akata and B95-8 had a stronger tropism for B cells than YCCEL1 or M81. These data suggest that different EBV strains will induce the development of lymphoid tumors with variable efficacy in immunocompromised patients and that there is a parallel between the cell tropism of the viral strains and the lineage of the tumors they induce. Thus, EBV strains can be endowed with properties that will influence their transforming abilities and the type of tumor they induce.

## INTRODUCTION

The Epstein-Barr virus (EBV) is a ubiquitous gammaherpesvirus that infects more than 90% of the adult human population. Although most EBV carriers do not display any clinical symptoms as the result of the viral infection, EBV is etiologically linked with up to 2% of all human malignancies, including multiple types of B-, T-, and NK-cell lymphomas, as well as carcinomas of the nasopharynx, the stomach, the parotid and the thymus [1, 2]. Although the virus infects the entire world human population, the geographic distribution of the tumors it causes is strikingly heterogeneous [3]. Burkitt’s lymphoma (BL) is endemic in Equatorial Africa and nasopharyngeal carcinoma (NPC) is one of the most common tumors encountered in South-East Asia [1]. Whilst EBV-associated T cell lymphomas are preferentially encountered in Japan, NK-cell tumors are mostly found in South East Asian individuals [1].

The factors that underlie these striking variations in disease incidence have long remained unclear, although the age at which EBV infection takes place, environmental factors such as food contaminations with nitrosamines and phorbol esters or smoking, and concurrent diseases such as malaria are all known to play an important role [4–6]. The genetic background of the affected individuals has also been invoked to explain this unusual phenomenon [7–10]. Yet another, non-exclusive hypothesis is that the occurrence of these diseases reflects the existence of multiple virus subtypes that are endowed with different properties but are found only in restricted geographic areas [11, 12].

We have previously reported that M81, an EBV strain isolated from a NPC that developed in a Chinese individual, spontaneously replicated in B cells *in vitro* and *in vivo* at unusually high levels and also had a high propensity to infect epithelial cells [13]. EBV lytic replication has been identified as a cancer risk factor as populations at risk for NPC evince high level of antibodies against viral lytic proteins [4, 14, 15].

These phenotypic traits are not shared by B95-8, a virus strain that has extensively been studied and that is genetically close to viruses found in Western countries where the incidence of NPC is low [12]. These observations demonstrate the existence of distinct EBV subtypes and suggest that the unusual properties evinced by M81-type viruses are likely to explain their tight association with NPC. Whilst the contribution of a subtype of EBV to NPC has been extensively studied, its implication in the development of gastric carcinoma (EBVaGC) has been comparatively neglected. The percentage of EBV-positive cases of gastric carcinomas is on average 10%, but can vary from 4 to 18% in different geographic areas and populations [16, 17]. The risk factors for the development of this tumor have not been clearly identified [18, 19]. In this paper, we report a comparative analysis of multiple EBV strains including three strains isolated from gastric carcinomas, with regard to their transformation abilities and cell tropism.

## RESULTS

### Generation of a panel of EBV strains, construction of a recombinant YCCEL1 virus and isolation of GP202

We collected a panel of EBV strains involved in different diseases and that infected individuals from different regions of the world (Supplementary Table 1). This panel included the recombinant viruses B95-8, Akata and M81. We also cloned the genome of the YCCEL1 virus from a gastric carcinoma cell line (Supplementary Figure 1A and 1B). The recombinant virus was stably transfected in 293 cells to generate a producer cell line that delivers high virus titers (Supplementary Figure 1C). In this recombinant virus, the F-plasmid is flanked by terminal repeats and is excised with high efficacy upon infection of B cells (Supplementary Figure 1D) [13]. Furthermore, we infected marmoset peripheral blood B cells with viruses rescued from SNU719 and GP202, 2 gastric carcinoma cell lines, to generate virus producer cell lines. GP202 was established from a gastric carcinoma that arose in a Portuguese patient and we performed an EBER staining to show that it is EBV-positive (Supplementary Figure 2A). Thus, it allows comparison with other gastric carcinoma viruses such as SNU719 and YCCEL1 that were isolated in Korean patients. Sequencing of the EBNA2 gene showed that GP202 is also a type A EBV strain (Supplementary Figure 2B and 2C).

### Different type A viruses differ in their ability to infect and transform B cells

**Figure 1:**
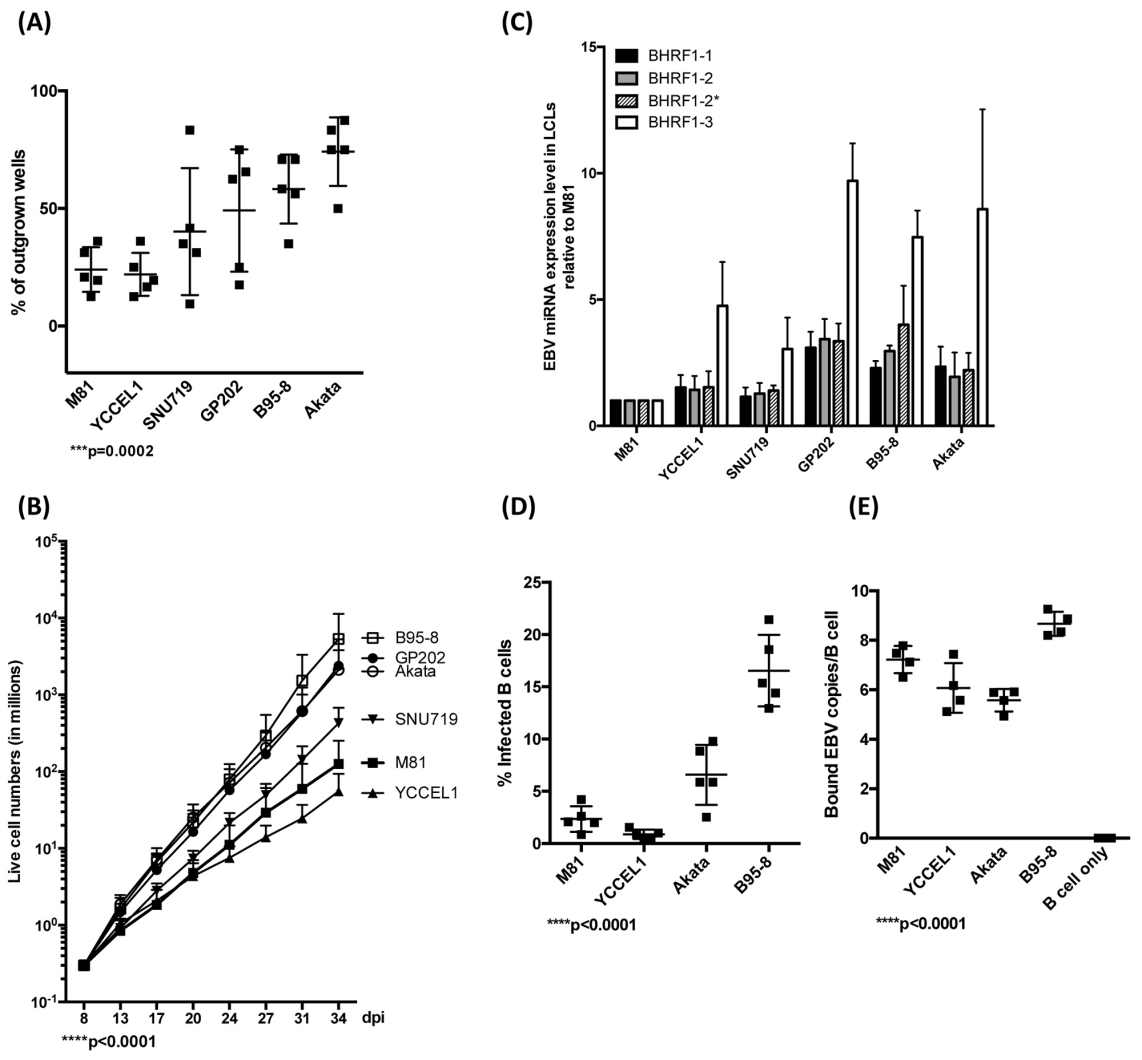
EBV strains transform B cells with variable efficiency **A**. This dot plot shows the efficiency of EBV-mediated transformation in infected primary B cell populations from 5 independent blood samples and seeded at 3 EBNA2-positive cells per well in 96 well cluster plates. The values given represent the percentage of outgrown wells. **B**. These curves show the increase in trypan blue- negative live cell numbers over time after infection of B cells from 7 independent blood samples with different EBV strains at high cell density. **C**. The bar graph shows the expression levels of all 4 members of the BHRF1 miRNA cluster in LCLs generated with 3 independent blood samples. **D**. We determined the efficiency of B cell infection by staining five different blood samples with an EBNA2-specific antibody 3 days after infection with the indicated viruses at the same multiplicities of infection as defined by qPCR. The graph gives the percentage of infected cells. **E**. The efficiency of B cell binding was assessed by qPCR after exposure of naïve B cells isolated from 5 independent blood samples to the same number of viral genomes. The dot plot shows the number of viruses bound per B cell. The p-values in (A), (B), (D), and (E) give the results of global test analyses by using global mixed linear model analyses with random effect. Error bars represent the mean with s.d.

We first compared the transforming potential of the virus panel. To this end, we infected primary B cells from 5 independent peripheral blood samples and performed transformation assays by seeding 3 or 30 EBNA2-positive B cells per well 3 days after infection and counted the number of outgrown wells 30 days post-infection (dpi) (Figure 1A and Supplementary Figure 3A). We found that these viruses formed 3 groups endowed with increasing transformation efficiency. YCCEL1 and M81 formed the first, SNU719 and GP202 the second and B95-8 and Akata the last group. We then infected 2*10E5 B cells in a bulk infection and assessed the growth rate of infected cells. The analysis of 7 blood samples showed results similar if not identical to the transformation assay (Figure 1B). M81 and YCCEL1 were found to be the least transforming viruses, GP202, Akata and B95-8 the most transforming ones. We quantified BHRF1 miRNA expression levels in the LCLs infected with the different viruses (Figure 1C). We found that the miRNA expression level globally markedly varies among the samples, with B cells infected with M81 expressing approximately five fold less BHRF1 miRNAs than B cells infected with B95-8 or GP202. The differences were most pronounced for miR-BHRF1-3 that was expressed approximately tenfold stronger in LCLs infected with GP202 than in those infected with M81. Interestingly, there was a positive correlation both between the transformation efficiency and the growth rate of LCLs and their BHRF1 miRNA expression levels (Figure 1, Supplementary Figure 3B and 3C). We also quantified the expression of some members of the BART miRNAs that are representative of the different BART miRNA clusters. However, it is important to note that B95-8 has a deletion that includes a large proportion of these miRNAs. There were some variations in expression levels, but with the exception of BART7*, these were less marked than those of the BHRF1 miRNAs and not statistically significant (Supplementary Figure 3D–3F). We noted a statistically significant inverse relationship between the levels of miR-BART7* and the growth rate of the different LCLs (Supplementary Figure 3D–3F). We pursued the characterization of B cell infection by performing binding assays and infection experiments. Here, we limited the analysis to the recombinant viruses to be able to compare viruses produced in cells of identical lineage. Akata and B95-8 infected B cells with a higher efficacy than M81 or YCCEL1. However, these differences could not be ascribed to a difference in binding, as Akata bound less efficiently to B cells than M81 (Figure 1D and 1E). Thus, M81 and YCCEL1 both transform and infect B cells with lower efficiency than Akata or B95-8.

We assessed latent gene expression in lymphoblastoid cell lines established after exposure to the virus panel (Supplementary Figure 4A to 4D). Here, we could not identify any striking differences between the viruses in terms of the level of protein expression, except in the higher LMP1 expression level in cells infected with SNU719 and YCCEL1 1 or 2 months post-infection (Supplementary Figure 4C). However, this was not a phenotypic trait common to all viruses associated with gastric cancer, as it was not found in cells infected with GP202 (Supplementary Figure 4) and it did not correlate with cell growth rate (Figure 1). Furthermore, we noted that the LMP1 protein was expressed at higher levels in LCLs transformed by M81, YCCEL1 and SNU719, one month after infection. At this time point, these 3 LCLs were also those that displayed the lowest cell growth rate, suggesting an inverse relationship between this process and LMP1 expression. This would fit with the observation that an increased LMP1 expression is toxic for the infected cells [20].

### Lytic replication in infected B cells

**Figure 2:**
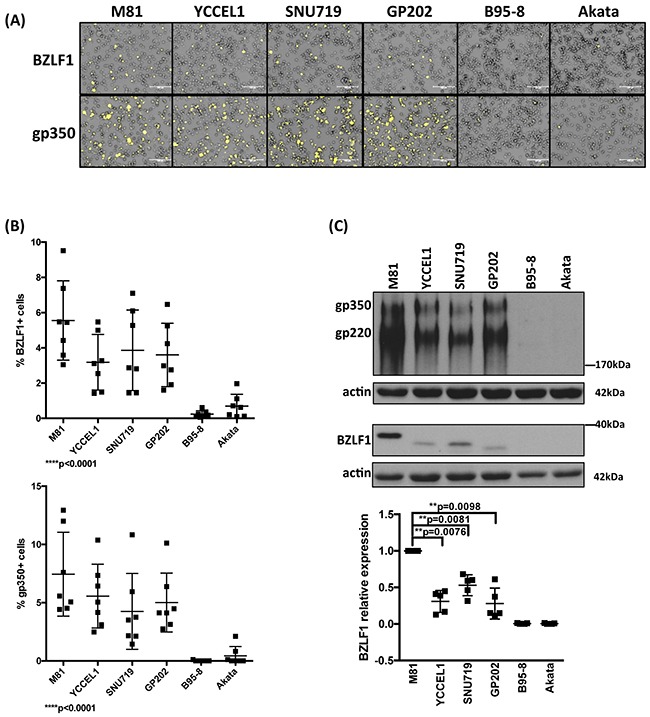
Lytic replication in LCLs infected with multiple EBV strains **A**. Representative BZLF1 and gp350 immunofluorescence stains performed on one set of LCLs generated with the same blood sample. **B**. The percentage of BZLF1- or gp350-positive cells in LCLs generated by the infection of 7 independent blood samples with 6 viruses. The 2 dot plots give the percentage of positive cells 4 weeks after infection. **C**. Representative BZLF1 and gp350-specific immunoblots performed on one set of LCLs generated with the same blood sample. Actin served as loading control. The 220kDa signals observed in the gp350-specific immunoblots are generated by a gp350 splice variant. The dot plot summarizes the BZLF1 protein levels observed after infection of five individual blood samples with the virus panel 1 month post-infection. The results are given relative to signals obtained with M81. The p-values shown in (B) give the results of global test analyses by using global mixed linear model analyses with random effect. In (C) we applied log-transformation of fold-changes for the analysis of normalized data without Bonferroni correction. Error bars represent the mean with s.d.

We then assessed the ability of LCLs generated with the virus panel to support lytic replication. We found that LCLs generated by M81 and YCCEL1 spontaneously initiated lytic replication around 18-20 dpi as shown by the detection of the immediate early lytic transactivator BZLF1, and of the late viral glycoprotein, gp350 (Figure 2A–2C). We obtained similar results after infection with SNU719 and GP202. In contrast, and as previously reported, we could hardly record any sign of lytic replication in LCLs transformed by B95-8 and only a few replicating cells after infection with Akata (Figure 2A–2C). The percentage of cells that expressed BZLF1 in LCLs transformed by YCCEL1, SNU719, or GP202, was on average approximately twofold smaller than the percentage of positive cells in LCLs transformed by M81 (Figure 2A and 2B). The total BZLF1 expression level in M81-infected cells assessed by western blot was 2 to 3 times stronger than after infection with gastric carcinoma viruses (Figure 2C). We extended our investigations to the gastric carcinoma cell lines from which the YCCEL1, SNU719 and GP202 viruses were isolated and found that they all exhibit spontaneous lytic replication at very low level (Supplementary Figure 2D).

### Different EBV strains induce tumor development *in vivo* with variable efficiency

**Figure 3:**
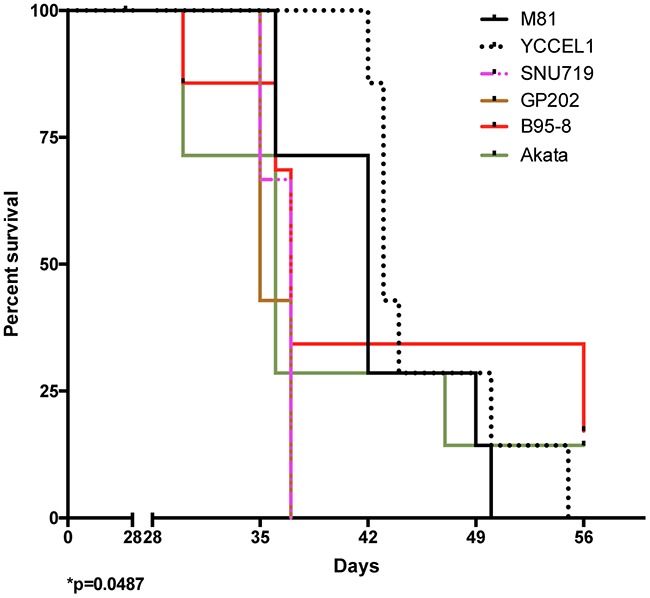
Survival in mice infected with various EBV strains The schematic shows Kaplan-Meier survival curves at multiple time intervals after infection with B cells exposed to one of 6 EBV strains. Each group included 7 mice. The Log-rank test shows a significant difference in survival (p=0.0487). Please also see the Supplementary Figure 5 and Supplementary Table 2.

We wished to determine whether the differences between viruses observed *in vitro* would extend to *in vivo* infections and injected primary B cells exposed to the different viruses into immunocompromised NSG mice. We monitored the mice for evidence of animal suffering (weight loss, apathy, signs of wasting, evidence of palpable tumor), upon which appearance the animals were euthanized. At autopsy, we found that all except two of these animals had macroscopic evidence of tumor growth mainly around the pancreas and to some extent also in the spleen, or in the liver (Supplementary Table 2). This allowed us to draw survival curves (Figure 3). We found that the groups of animals infected with Akata or B95-8 showed the earliest signs of tumor growth, approximately 30 dpi. This is consistent with the observation that these viruses induce B cell growth *in vitro* with the highest efficiency. Members of these 2 groups of mice continued to develop tumors over a period of 30 days after identification of the initial cases. However, 2 mice remained free of tumor infiltration at termination of the experiment. Mice infected with GP202 or SNU719 showed evidence of tumor development one week later than those infected with B95-8 or Akata. However, in contrast to these latter mice, all the animals infected with GP202 or SNU719 showed evidence of tumor growth within a few days. Mice infected with M81 or YCCEL1 started developing tumors even later and continued to do so over a period of 3 weeks. All animals belonging to these 2 groups were eventually found to carry a lymphoid tumor.

Histological evaluation of the tumors showed expression of the EBNA2 and LMP1 latent genes in all cases. Although IHC is not a quantitative method, LMP1 expression levels were heterogeneous after infection with the different types of viruses (Supplementary Figure 5). Here we found that M81 and SNU719 expressed LMP1 at a higher level than the other viruses, an observation that only partly reflects our results obtained *in vitro*. Staining of tumors for lytic markers showed evidence of replication in M81, GP202, SNU719 and YCCEL1 (Supplementary Figure 5). Replication was strongest in animals infected with M81. Mice infected with Akata showed some evidence for lytic replication, although at levels that were much weaker. Infection with B95-8 showed hardly any evidence of replication.

### YCCEL1 infects human primary epithelial cells derived from a respiratory epithelium, but less efficiently than M81

**Figure 4:**
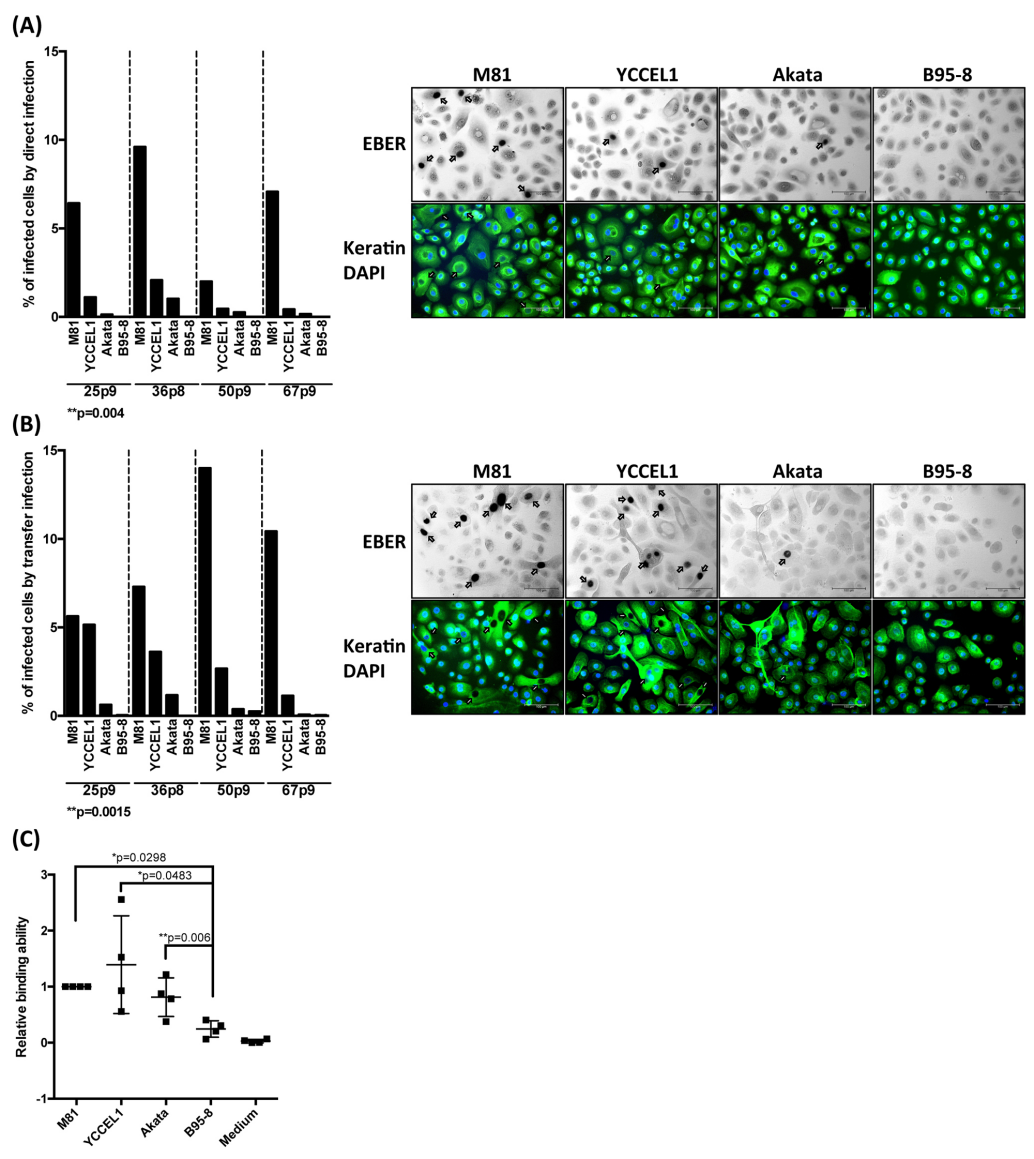
Infection of primary cells from a respiratory epithelium with multiple EBV strains **A**. We infected four different batches of primary cells from the respiratory epithelium with supernatants containing the same numbers of infectious particles from the indicated EBV strains and stained them for EBER expression 3 days post-infection. The cells were also submitted to an immunostain with an antibody specific for keratin and the nuclei were counterstained with DAPI. The percentage of infected cells is given in the adjacent graph of bars and we show the results of 4 infections performed with samples from independent individuals. The arrows point to EBER-positive cells. **B**. Same experiment as in (A) except that we used transfer infection. **C**. The primary samples used in (A) and (B) were exposed to viral supernatants. After extensive washings, we quantified the number of copies bound to the target cells. The results are given relative to signals obtained with M81. The p-values shown in (A) and (B) give the results of global mixed linear model analyses with random effect. In (C) we applied log-transformation of fold-changes for the analysis of normalized data without Bonferroni correction. Error bars represent the mean with s.d.

Although both YCCEL1 and M81 were isolated from carcinomas, the lineage of the tumors they caused was different. Nasopharyngeal carcinoma is typically an undifferentiated tumor that develops from the respiratory epithelium that covers the nasopharynx [21]. Gastric carcinomas are also frequently poorly differentiated but develop from the gastric glandular epithelium [21]. We assessed the infection spectrum of both types of viruses first by infecting primary cells obtained from the respiratory epithelium that covers the sphenoidal sinus and shows the same differentiation as the nasopharyngeal epithelium [22]. These human primary epithelial cells were subjected to infection with YCCEL1, B95-8, Akata and M81. We used both direct infection in which epithelial cells are exposed to cell-free virus supernatants or transfer infection in which virus is first loaded on resting B cells and the infected B cells are admixed with epithelial cells as previous described [23]. The percentage of EBV-infected primary epithelial cells was visualized by an EBER *in situ* hybridization and the results of both types of infections are given (Figure 4A and 4B). These experiments demonstrated that YCCEL1 infects human primary squamous epithelial cells both through direct and transfer infection, although the infection rate was generally approximately fivefoldlower than with M81 that has previously been found to be highly epitheliotropic [13]. In all these experiments, B95-8 barely infected the cells, as we reported previously [13] and Akata was between 10 and 100 times less infectious than YCCEL1. A binding assay performed with these viruses and with human primary epithelial cells showed that YCCEL1, M81 and Akata bind to epithelial cells more efficiently than B95-8 (Figure 4C). In conclusion, YCCEL1 can also be classified as an epitheliotropic EBV strain, albeit less potent than M81.

### YCCEL1 infects human gastric carcinoma cell lines efficiently

**Figure 5:**
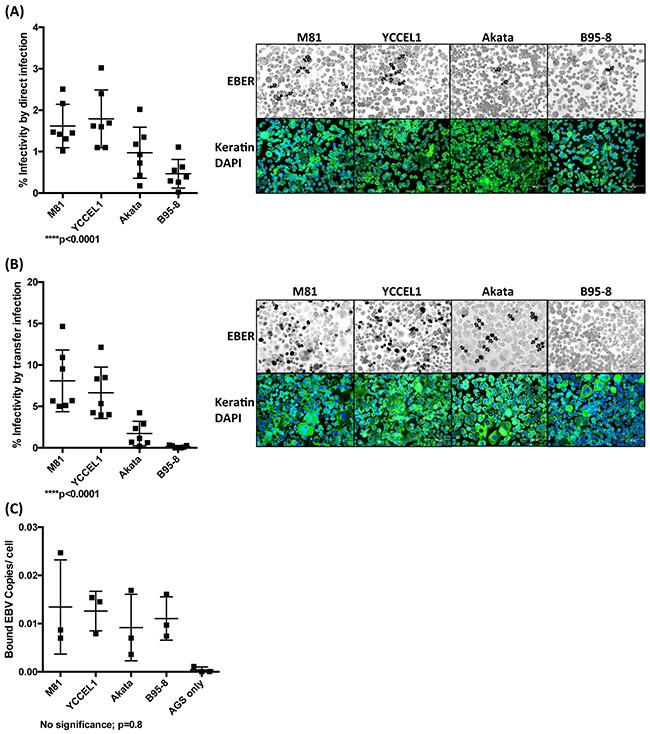
EBV strains infect AGS cultures with variable efficiency **A**. We infected the AGS cell line with different EBV strains at the same multiplicities of infection and performed an EBER *in situ* hybridization 3 days post-infection, coupled to an anti-keratin immunostain. We counted the percentage of EBV-infected cells and give the results from 7 independent experiments in a dot plot. We also show a representative example of these infections. The arrows point to EBER-positive cells. **B**. Same as in (A) but using transfer infection. **C**. The results of a binding assay with viral supernatants used in (A) and (B) and the AGS cells. The results are given as the number of EBV copies bound per cell. Here we show the results from 3 independent experiments. The p-values show in (A) to (C) give the results of global test analyses using global mixed linear model analyses with random effect. Error bars represent the mean with s.d.

We then performed similar experiments with a panel of epithelial cell lines established from carcinomas of the stomach and of the colon (SNU-638, MKN45, IPA220, NCI-N87, Caco2). We found very low infection rates in MKN45, NCI-N87 and IPA220 (less than 1/10000) after transfer infection with M81 and YCCEL1 (Supplementary Figure 6). SNU-638 and Caco2 were completely resistant to infection. However, AGS, an EBV-negative gastric adenocarcinoma cell line established from a Caucasian patient could be infected at a significant rate, as previously reported (Figure 5) [24–26]. Direct infection of AGS cells with M81 and YCCEL1 gave rise to similar infection rates and both viruses were clearly more infectious than Akata or B95-8 (Figure 5A). The picture remained similar after transfer infection with these viruses, although in that case M81 was more infectious than YCCEL1 (Figure 5B). However, the variable tropism of these EBV strains for AGS cells cannot be ascribed to different binding abilities of the virus strains to this cell line (Figure 5C).

**Figure 6:**
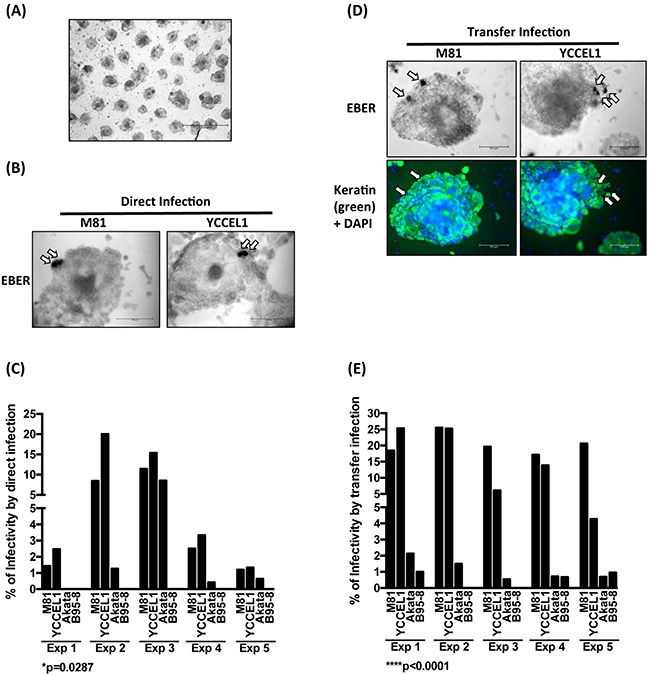
YCCEL1 efficiently infects gastric spheroids **A**. The picture shows the characteristic morphology of gastric spheroids generated by passaging AGS cells *in vitro* on a Matrigel matrix (Scale bar 1 mm). **B**. Representative pictures of gastric spheroids exposed to virus supernatants containing M81 and YCCEL1 and subjected to *in situ* hybridization with an EBER-specific probe (Scale bar 100 μm). The arrows point to EBER-positive cells. **C**. This bar graph compiles the results of 5 independent direct infection experiments of gastric spheroids with four different viruses at the same multiplicity of infection. We calculated the percentage of gastric spheroids that contained EBER-positive cells. **D**. We repeated the experiments described in (B) but using EBV transfer infection. We combined the detection of EBERs with a keratin staining to ensure that the infected cells are of epithelial lineage. Two representative examples are shown (Scale bar: 100 μm). Arrows show the EBER-positive cells. **E**. The transfer infections of gastric spheroids were repeated 5 times and their results were plotted in the presented graph. The p-values show in (C) and (E) give the results of global test analyses using global mixed linear model analyses with random effect.

We then turned to a more physiological model of infection in which 3D gastric structures are infected. AGS cells can be simulated by growth factors present in the Matrigel matrix to build gastric spheroids *in vitro* (Figure 6A) [27]. Upon stimulation, the cells built regular hollow spheres that crudely reproduce the architecture of gastric glands. We infected these structures by direct and transfer infection, fixed them and performed EBER staining, coupled to keratin immunostains. We could detect EBER-positive cells in the epithelium after both types of infection (Figure 6B and 6D). Infection rates were highest after direct infection with YCCEL1, closely followed by M81 (Figure 6B and 6C). Infections with Akata and B95-8 were generally much less efficient (Figure 6B and 6C). Transfer infection yielded similar results, albeit M81 generally infected these structures more efficiently than YCCEL1 (Figure 6D and 6E).

### The tropism for epithelial cells is governed by the expression of a viral protein that fuses with the cell membrane

**Figure 7:**
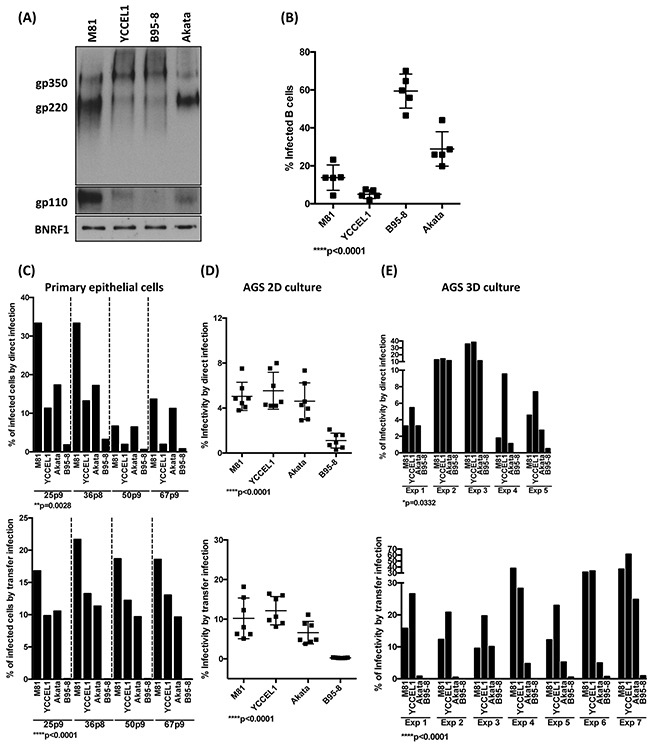
The level of gp110 within EBV viral particles partially regulates the viral tropisms toward its hosts **A**. Immunoblots of viral glycoproteins gp350/220 and gp110 in pelleted viral particles. The tegument protein BNRF1 served as a loading control. **B**. The dot plot shows the percentage of infected B cells determined by an EBNA2-specific staining 3 dpi at the same MOI after complementation of the indicated viruses with gp110. The results were compiled from infection of five independent blood samples. **C, D, E**. We repeated the infection experiments of primary epithelial cells, 2D AGS and 3D AGS cultures described in Figure 4, 5 and 6, respectively, using gp110-complemented viruses. The top panels show the results of direct infections, the bottom panels those of transfer infections. The p-values show in (B) to (E) give the results of global test analyses by using global mixed linear model analyses with random effect. Error bars represent the mean with s.d.

EBV infection of its target cells is mediated by the interaction of viral glycoproteins with the surface of the target cells [28]. We assessed the expression of two of the most abundant viral glycoproteins gp110 and gp350 by western blot and found that the expression of gp350 was roughly similar in all 4 recombinant viruses, although M81 and Akata expressed more of gp220, a splicing form of gp350 (Figure 7A). However, the gp110 expression level was clearly higher in M81 than in the other viruses (Figure 7A). Therefore, we repeated the infection experiments described above with viruses complemented with high level of the surface viral glycoprotein gp110 by overexpressing the BALF4 gene during viral production. This complementation leads to an increase in gp110 content in the virions [29]. GP110^high^ virions were previously found to be much more infectious than gp110^low^ virions [29]. Complementation with gp110 doubled the efficiency of infection in human primary B cells with all virus strains without changing the overall picture (Figure 7B and 1C). Infection of primary human respiratory epithelial cells showed that M81 remains the most infectious virus, even after gp110 complementation. However, infection with Akata and YCCEL1 yielded results that were much more similar than in the absence of complementation, both in direct and transfer infections (Figure 7C, Supplementary Figure 7). Similar results were obtained with infection of 2D AGS where direct infection with Akata became much closer to M81 and YCCEL1 than without complementation (Figure 7D, Supplementary Figure 7). This effect became less visible for the 3D AGS cultures, where complementation of gp110 did not change the overall picture, with YCCEL1 and M81 remaining more infectious than the other two viruses (Figure 7E).

## DISCUSSION

In this paper, we analyzed the phenotypic characteristics of 6 different EBV isolates.

This analysis showed that type A viruses comprise viruses with variable properties. Whilst B95-8, Akata and GP202 induced cell growth more efficiently than YCCEL1, SNU719 and M81, all strains except Akata and B95-8 induced lytic replication at high levels in infected B cells. The variable transformation efficiency of these isolates identified by *in vitro* studies was also visible to a large extent in immunocompromised mice and suggests that different EBV strains will induce the development of lymphoid tumors with variable efficacy in immunocompromised patients. Whilst YCCEL1 and M81 infected primary epithelial cells, gastric carcinoma cells and gastric spheroids more efficiently than Akata or B95-8, these two viruses had a stronger tropism for B cells than the other isolates, suggesting that there is a parallel between the cell tropism of the viral strains and the lineage of the tumors they induce.

Owing to difficulties in propagating EBV in culture, only a few viral strains can be produced in large amounts *in vitro* to perform infection experiments. We have now cloned YCCEL1, a virus isolated from a gastric carcinoma cell line to produce large amounts of the virus. A recent publication has reported the cloning of YCCEL1 and SNU719, although there is still limited information on the characteristics of these clones [30]. In contrast to our recombinant YCCEL1 clone, these recombinant viruses carry the F-plasmid within the viral genome, which required the insertion of F-plasmid between the non-coding region of BVRF1 and BVRF2 [30]. This might potentially alter the gene expression pattern in the neighborhood of the insert that is transcribed in both directions [13, 31]. Our YCCEL1 recombinant excises the F-plasmid upon replication, leading to the reconstitution of an intact genome devoid of foreign sequences. We have also isolated a new EBV strain from a gastric carcinoma cell line that was established from the tumor of a European patient. This will allow a comparison with YCCEL1 and SNU719 and the identification of phenotypic traits that reflect geographic rather than tumor type-specific differences.

The first set of experiments performed in B cells showed large differences in the transforming abilities of the viruses *in vitro* and *in vivo*. B95-8, Akata and GP202 transformed B cells more efficiently that the 3 other strains. This led to the production of 400 times more B cells within 34 days after transformation with B95-8 than with YCCEL1. This property was independent of the isolation of viruses from epithelial or from lymphoid cells and was also independent of the Western or Asian origins. Furthermore, B95-8 and Akata infected B cells more efficiently than M81 and YCCEL1, and this property was independent of the ability of these viruses to bind to their target. Taking into account both the efficiency of B cell infection and the transformation efficiency, B95-8 and Akata would lead to B cell expansion much more efficiently than M81 and YCCEL1. Such a divide across EBV isolates was previously recognized for a subgroup of viruses that were referred to as type B viruses [32, 33]. However, all viral strains included in the present study are type A viruses. It is noteworthy that there is a positive correlation between the transforming abilities of the viruses and the level of BHRF1 miRNA expression they induce in infected cells. This is in line with the properties of these viral miRNAs [34]. This suggests the existence of additional subgroups within type A viruses, characterized by a variable tropism for B cells and variable transformation efficiencies possibly due to a higher BHRF1 miRNA expression. This has important consequences for the development of EBV-driven lymph proliferations in immunocompromised patients. More transforming viruses might be prone to cause more tumors than less transforming ones. The results of the infection experiments in immunocompromised mice partially support this hypothesis, as infection with B95-8 and Akata, and to some extent GP202 and SNU719, cause tumors more rapidly than with M81 or YCCEL1. However, it is important to note that there was some heterogeneity among the groups of mice infected with all types of viruses, except GP202 and SNU719 that caused tumor development within a narrow period of time. One out of 7 mice infected with Akata or B95-8 even managed to keep the number of infected B cells low. This suggests that other properties of the virus, e.g. the ability to withstand apoptosis, might also influence the outcome of infection.

We then conducted a series of experiments that aimed at defining the infectious spectrum of the different viruses. We also found significant differences between YCCEL1, M81, B95-8 and Akata. The former 2 viruses infected epithelial cells with much higher efficiency than B95-8 and Akata. However, both viruses also showed differences, with M81 being slightly more infectious for primary epithelial cells from the respiratory epithelium. Reciprocally, YCCEL1 showed a slightly stronger tropism for gastric spheroids upon direct infection. Although EBV can penetrate into epithelial cells following mechanical disruption of the epithelial layer as seen in tissue wounds, our present data confirm that some EBV strains have the ability to infect epithelial cell directly or through transfer infections[13, 35]. It is important to note that YCCEL1 had a stronger tropism for gastric epithelial cells, and to some extent for primary respiratory cells than Akata or B95-8. This observation is consistent with the observation that YCCEL1 was isolated from a gastric carcinoma and reinforces the view that the ability of EBV strains to cause cancer is linked with their infectious spectrum.

Why are the molecular mechanisms that explain the variable infectious spectra of different EBV strains? We initiated some work in that direction by assessing the expression pattern of two major glycoproteins within the group of virions. We found that gp110 is expressed at lower levels in YCCEL1, Akata and B95-8 than in M81. Overexpression of gp110 in the 4 virus types markedly increased the infection rates in epithelial cells for all tested viruses. However, the relative efficiencies of infection did not change for M81, YCCEL1 and B95-8, the latter virus remaining less infectious than the former two. This shows that that additional factors than gp110, for example, other viral glycoproteins, also influence the tropism of different EBV strains. Interestingly, transfection of gp110 into Akata producer cell lines led to substantial increases in the infection rates of primary epithelial cells, suggesting that the relative deficiency in gp110 in Akata virions explains the lower infection rates observed with wild type Akata. However, this effect could not be observed with the gastric spheroids. In that case, Akata virions remained less infectious, even after gp110 overexpression.

We found that all EBV strains isolated from gastric carcinomas we tested caused spontaneously lytic replication in B cells infected *in vivo* and *in vitro*, and in infected gastric carcinoma cell lines, a property reminiscent of M81 [13]. This fits with the recent description of highly abundant EBV lytic transcripts both in primary EBV-positive gastric carcinoma samples and in EBV-positive cell lines [36, 37]. It is also concordant with our observation that cell lines derived from EBV-positive carcinomas express lytic proteins. Moreover, a large scale epidemiological study performed on a cohort of Indian patients with gastric diseases concluded that the level of BZLF1 and the other lytic transcripts in the blood are significantly associated with gastric cancer [38]. BARF1, an early lytic protein also highly expressed in EBV-positive GC and NPC samples has been found to contribute to the growth of epithelial cells [39]. Thus, EBV lytic replication might not only be a risk factor for NPC but might also contribute to the development of gastric carcinoma. Some lytic proteins such as BZLF1 and EBV lytic transcripts are endowed with transforming properties and repeatedly induced lytic replication in an EBV-positive NPC cell line promoted chromosomal instability [40, 41]. Whether lytic replication contributes to both EBV-associated carcinomas and why some EBV strains initiate spontaneous lytic replication in the infected hosts more efficiently than others deserves further investigation.

## MATERIALS AND METHODS

### Ethics statement

All human primary B cells used in this study were isolated from anonymous buffy coats purchased from the Blood Bank of the University of Heidelberg. No ethical approval is required. All human primary epithelial cells in this study were obtained from normal sphenoidal sinus cells during hypophysectomy. The Ethics Committee of the University of Heidelberg approved the study (Approval 392/2005) [22]. All animal experiments were performed in strict accordance with German animal protection law (TierSchG) and were approved by the federal veterinary office at the Regierungspräsidium Karlsruhe, Germany (Approval number G156-12). The mice were housed in the class II containment laboratories of the German Cancer Research and handled in accordance with good animal practice with the aim of minimizing animal suffering and reducing mice usage as defined by Federation of European Laboratory Animal Science Associations (FELASA) and the Society for Laboratory Animal Science (GV-SOLAS).

### Cell lines

HEK293 human embryonic kidney cells [42] were purchased from ATCC and used for stable transfection of EBV-BACMIDs. YCCEL1 and SNU719 are gastric carcinoma cell lines established from Korean patients [43, 44]. GP202 and IPA220 are gastric carcinoma cell lines established from Portuguese patients [45]. Other gastric carcinoma cell lines included AGS (ATCC: CRL-1739); MKN45 [46]; SNU-668 (Korean Cell Line Bank: 00638); NCI-N87 (ATCC: CRL-5822). Caco2 (ATCC: HTB-37) is a colorectal adenocarcinoma cell line originated from a Caucasian patient. Lymphoid producer cell lines were generated by infection of purified B cells from marmoset monkeys with YCCEL1, SNU719 or GP202 viruses. We also used human CD19-positive primary B lymphocytes isolated from buffy coats to establish permanently growing lymphoblastoid lines (LCL) with various viruses. All cells were routinely cultured in RPMI-1640 medium (Invitrogen) supplemented with 10% fetal bovine serum (FBS) (Biochrom), whilst primary B cells were supplemented with 20% FBS until establishment of LCLs. Primary epithelial cells isolated from normal sphenoidal sinus biopsy materials were maintained in keratinocyte serum-free growth medium (KGM-SFM; Invitrogen) as described previously [22].

### Recombinant viruses

The EBV strains M81, B95-8 and Akata are available as recombinant BACMIDs for which producer cell lines based on 293 cells have been generated [13, 47, 48]. We cloned the YCCEL1 DNA by transfecting a plasmid that contain a prokaryotic F-plasmid flanked by the EBV terminal repeats at both ends and carrying the chloramphenicol (Cam) resistance gene, the gene for green fluorescent protein (GFP) and the (Hygromycin) Hyg resistance gene (B1073) into a marmoset LCL generated with YCCEL1. Cells that successfully underwent recombination between the F-plasmid and the YCCEL1 genome were selected by adding hygromycin to the culture media (100 μg/ml). The hygromycin-resistant cells were further expanded and the circular EBV genomes present in these cells were extracted by using a denaturation-renaturation method [47] and transferred into the *E.coli* strain DH10B by electroporation (1000V, 25μF, 200 Ohms). The transformed *E.coli* clones were further assessed by restriction enzyme analysis of plasmid minipreparations. The YCCEL1 BACMID was stably transfected into 293 cells as described previously [47]. To assess the genome integrity of recombinant EBV within the stable clones, the circular EBV genomes were extracted from cells and transferred into the *E.coli* strain DH10B and the expanded plasmids in bacteria were further assessed by restriction enzyme analysis of plasmid minipreparations.

### Virus production and quantification

To produce infectious viruses, 293 cells stably transfected with recombinant EBV-BACs were transfected with an expression plasmid encoding the BZLF1 gene (p509) using the liposome-based transfectant Metafectene (Biontex). We also transfected the EBV-positive gastric cell lines GP202, YCCEL1 and SNU719 with BZLF1 to collect infectious viruses that were used to generate the marmoset producer LCLs. To evaluate the effects of gp110 overexpression on infection, we co-transfected an expression plasmid that encodes this gene (pRA) with BZLF1 into the producer cell lines. Three days after transfection, virus supernatants were collected and filtered through a 0.4 μm filter. We also collected supernatants from the marmoset LCLs generated with the YCCEL1, GP202 and SNU719 viruses.

Quantification ofEBV equivalents per milliliter of supernatant was performed by quantitative real-time PCR analysis (qPCR) as described previously [49].

### B cell infections

Purified CD19+ human B cells were exposed to viral supernatant with various multiplicity of infection (MOI), determined by qPCR, for two hours, then washed once with PBS and cultured with RPMI supplemented with 20% FBS in the absence of immunosuppressive drugs. For transformation assays, the percentages of EBNA2-positive cells of sample were evaluated by immunostaining 3 days post-infection (dpi). Cell numbers corresponding to 3 or 30 EBNA2-positive cells per well were seeded into 48 wells of 96-U-well plates that contained 10^3^ gamma-irradiated WI38 feeder cells. Non-infected B cells served as a negative control. The outgrowth of LCLs was monitored at 30 dpi.

### Quantification of EBV viral miRNAs

The quantification of viral miRNAs was previously described [50, 51]. Briefly, EBV viral miRNAs were reverse transcribed from total RNA extracted from LCL cells with Trizol using specific stem-loop primers and TaqMan miRNA reverse Transcription Kit (Applied Biosystems). Reverse transcription and amplification of the cellular snoRNA RNU48 was performed in parallel to normalize for cDNA recovery (Assay ID 001006; Applied Biosystems). Real-time PCR was performed on an ABI STEP ONE PLUS Sequence Detection System (Applied Biosystems).

### Epithelial cell infections

Viruses used for direct infection of primary epithelial cells were concentrated by centrifugation of infectious supernatants (20,000 x g for 2 hr at 4°C) and resuspended in PBS. The viruses were applied to primary epithelial cells at a multiplicity of infection of 200 viral genomes per cell for 72 hrs. For transfer infection, primary B cells were exposed to viral supernatants at a MOI of 100 for 2 hr at room temperature and left in RPMI-20% FBS for 20hr in a CO_2_ incubator [23]. Virus-loaded B cells were then washed once in culture medium used for primary epithelial cells (KGM-SFM) and cocultured with the primary epithelial cells at a concentration of 3 virus-loaded B cells per epithelial cell. The B cells were carefully removed 24 hr post-seeding and the infection rate of the primary epithelial cells was determined 48 hr thereafter by *in situ* hybridization with an EBER-specific PNA probe in conjunction with a PNA ISH detection kit (Dako) according to the manufacturer’s protocol. The infection experiments with the AGS cell line were performed according to the same experimental protocol, except that AGS cells were maintained in RPMI-10% FBS. We infected the cells either with unmodified viruses or with viruses trans-complemented with gp110. Infections with or without gp110 were performed in parallel.

### Binding assays

Supernatants containing viruses were treated with 15 μg DNase I (Roche) per ml at 37°C for 1 hour to digest free viral DNA. We performed binding assays by co-incubating human primary B cells with viruses at a MOI of 10. Primary epithelial cells or AGS cells were trypsinized and exposed to viruses at a MOI of 200 at 4°C for 2 hours on a rolling device. EDTA was added to the supernatants (20 mM final concentration) to inactivate the DNase I and the cells were washed 3 times with ice-cold PBS. The washed cells were subjected to genomic DNA extraction using a DNeasy Blood and tissue kit (Qiagen). We quantified the number of EBV copies per ng of the isolated genomic DNA using qPCR.

### Antibodies

Mouse monoclonal antibodies against BZLF1 (BZ.1), gp350/220 (72A1), EBNA2 (PE2) and a Cy-3-conjugated goat-anti-mouse secondary antibody (Dianova, Invitrogen) were used for immunofluorescence staining. We performed western blots with mouse monoclonal antibodies against BZLF1 (BZ.1), gp350 (OT6), LMP1 (CS1-4), EBNA2 (PE2), EBNA3C (A10), rat monoclonal antibodies against EBNA1 (2B4), LMP2A (4E11), EBNA3A (E3AN-4A5), EBNA3B (6C9). Mouse monoclonal antibodies specific to BZLF1 (Clone BZ.1), gp350 (Clone OT6) and LMP1 (CS1-4) were used for immunohistochemical staining against EBV proteins in infected murine tissues.

### Immunofluorescence staining

The cells were fixed with 4% paraformaldehyde in PBS for 20 min at room temperature. Fixed cells were permeabilized in PBS 0.5% Triton X-100 for 2 min except for samples stained with an antibody specific for the viral glycoprotein gp350. Cells were incubated with the first antibody for 30 min at 37°C, washed in PBS three times, and incubated with a secondary antibody conjugated to Cy-3 for 30 min at 37°C. The stained slides were embedded in 90% glycerol and stored at 4°C.

### Western blot analysis

Proteins were extracted with a standard lysis buffer (150 mM NaCl, 0.5% NP-40, 1% Sodium deoxycholat, 0.1% SDS, 5 mM EDTA, 20 mM Tris-HCl pH7.5, proteinase inhibitor cocktail (Roche)) for 15 min on ice followed by sonication to shear the genomic DNA. Up to 20μg of proteins denatured in Laemmli buffer for 5 min at 95°C were separated on SDS-polyacrylamide gels and electroblotted onto a nitrocellulose membrane (Hybond C, Amersham). Protein extracts generated for the analysis of viral glycoproteins were prepared in Laemmli buffer without 2-mercaptoethanol to avoid reducing conditions. After pre-incubation of the blot in 3% milk dry powder in PBST (PBS with 0.2% Tween 20), the antibody against the target protein was added and incubated at room temperature for 1 hr. After extensive washings in PBST, the blot was incubated for 1 hr with suitable secondary antibodies coupled to horseradish peroxidase (goat anti-mouse (Promega), goat anti-rabbit (Life technologies), or rabbit anti-goat (Santa Cruz) IgG). Bound antibodies were revealed using the ECL detection reagent (Pierce).

### Infection experiments in NSG mice

We isolated human CD19+ B cells from buffy coats and exposed them to virus supernatants for 2 hours at room temperature under constant agitation at a MOI sufficient to generate 20% of EBNA2-positive cells. The infected cells were collected by centrifugation and washed twice with PBS. 2*10ˆ6 infected primary B cells, equivalent to 4*10ˆ5 infected cells, were injected intraperitoneally into NSG mice (*NOD.Cg-Prkdc^scid^Il2rg^tm1Wjl^/SzJ*; NSG). The pre-established inclusion criteria in this study were healthy male NSG mice aged between 6 and 8 weeks. The mice were euthanized as soon as any clinical symptoms appeared (apathy, food refusal, ruffled hair, weight loss, palpable tumor). After careful autopsy, the organs were subjected to macroscopic and microscopic investigation, including hematoxylin and eosin (H&E) staining and immunohistochemistry.

### Immunohistochemistry

The organs from the studied mice were fixed in 10% formalin overnight and embedded in paraffin blocks. 3-μm-thin continuous sections were prepared and submitted to antigen retrieval at 98°C for 40 min in a 10 mM sodium citrate, 0.05% Tween 20 pH 6.0 solution. Bound antibodies were visualized with the Envision+ Dual link system-HRP (Dako). In parallel, adjacent sections were stained with H&E. The presence of EBV was detected by *in situ* hybridization with an EBER-specific PNA probe, in conjunction with a PNA detection kit (Dako) following the manufacturer’s protocol. Pictures were taken with a camera attached to a light microscope (Axioplan, Zeiss).

### Generation of gastric spheroids and EBV infection

50μl of Matrigel (BD Bioscience) thawed on ice at 4°C overnight was added to each well of a 8-well chamber glass culture slide. After solidification at 37°C for 30 min, 5,000 AGS cells in culture medium were loaded on the top of the Matrigel matrix. The well-formed sphere-shaped gastric spheroids formed by AGS cells can be observed around 6 days post-seeding. At this point, virus infections were performed by direct exposure to virus supernatants or by B cell transfer infection as described for primary epithelial cells. Three days after infection, the Matrigel and the infected spheroids were air dried and the cells were subjected to EBER *in situ* hybridization. We further stained the samples subjected to transfer infection with an antibody specific for keratin to ascertain that the EBER-positive cells were of epithelial lineage.

### Statistical analysis

We applied mixed linear model analyses with random effect for the data reported as absolute values without further normalization. We show the p value of the global test at the left-bottom side of each figure. We applied log-transformation of fold-changes for the analysis of normalized data. The survival curves of the infected animals were evaluated with a Kaplan-Meier Plot and took into account animals that did not show tumors at autopsy. Global test statistics was applied to experiments performed with 4 or more different EBV strains, in order to evaluate the extent of phenotypic variations induced by infection with different EBV strains. The p-values of this global test are given at the left-bottom side of each figure and p-value less than 0.05 indicate statistically significant variation between the EBV strains. We calculated Pearson *r* correlation coefficients and their accompanying p-values to evaluate the degree of correlation between EBV-mediated transformation efficiency or the proliferation rate of EBV-infected B cells with the expression levels of EBV viral miRNAs. The statistical analyses were performed with the SAS 9.3 and GraphPad Prism 5 software and were drawn with GraphPad Prism 5 software.

## SUPPLEMENTARY FIGURES AND TABLES


