# Supplementary material for: The biological properties of different Epstein-Barr virus strains explain their association with various types of cancers
Source: Oncotarget. 2016 Dec 30;8(6):10238–54. doi: 10.18632/oncotarget.14380 (PMC5354655; doi:10.18632/oncotarget.14380)
Supplement: Supplementary file 1 [file oncotarget-08-10238-s001.pdf]

The biological properties of different Epstein-Barr virus strains explain their association with various types of cancers

SUPPLEMENTARY FIGURES AND TABLES

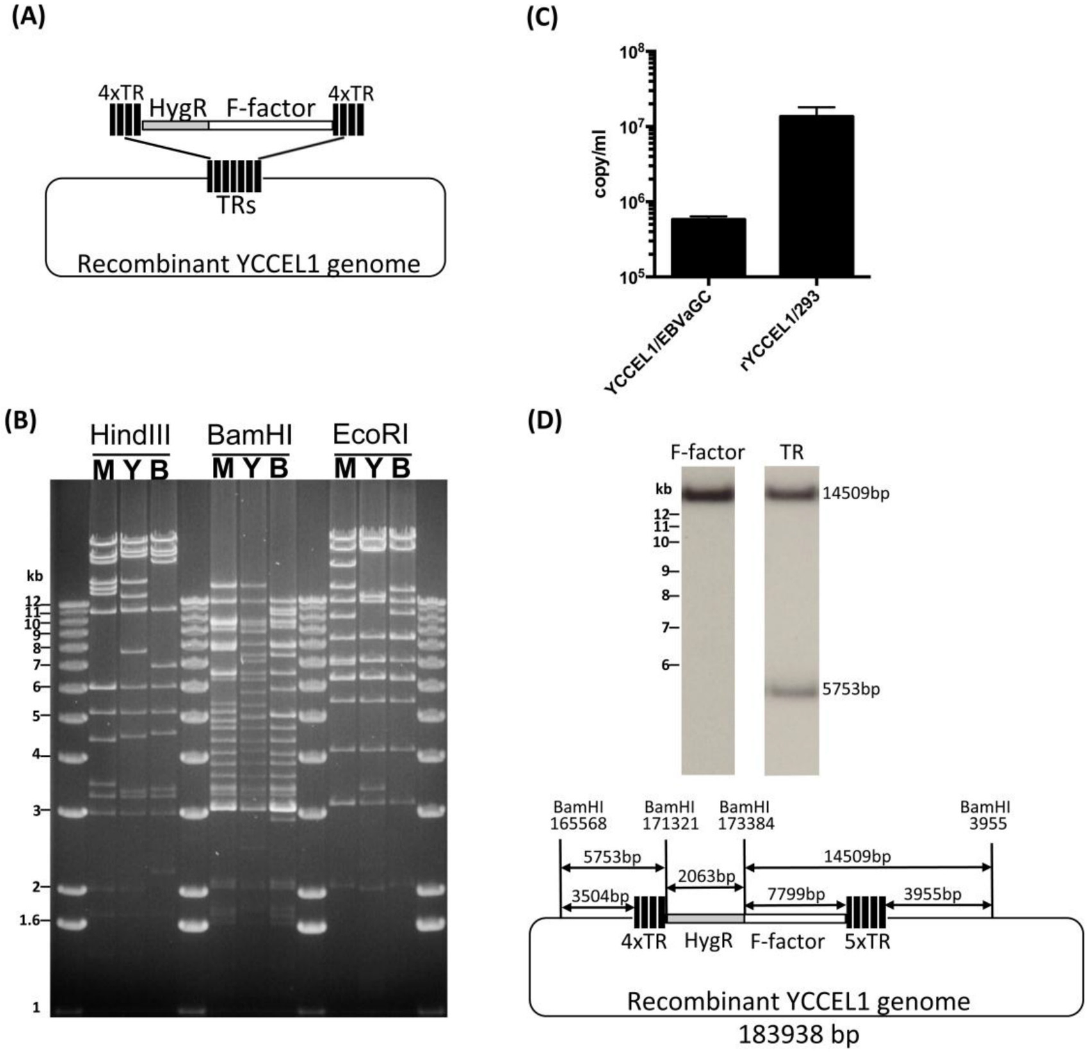

Supplementary Figure 1: Construction of the recombinant YCCEL1 EBV genome. A. We cloned the YCCEL1 genome by inserting an F-factor plasmid coupled to a hygromycin resistance cassette into the viral genome. The F-factor was flanked by 4 terminal repeats (TR) at both ends to allow homologous recombination. The schematic shows the structure of the targeting vector and the position of the inserted F-plasmid within the YCCEL1 genome. **B.** Restriction pattern of the recombinant YCCEL1 (Y), M81 (M) and B95-8 (B) viruses. Minipreparations of plasmid DNA were cleaved with the HindIII, BamHI, or EcoRI restriction enzymes. **C.** The parental YCCEL1 cell line and the YCCEL1/293 cells were transfected with a BZLF1-expressing plasmid. We quantified viral titers in supernatants 3 days post-transfection by qPCR. **D.** The recombinant YCCEL1 viral DNA was cleaved with BamHI, separated on an agarose gel and subjected to a Southern blot analysis with a probe specific for the EBV terminal repeats or for the F-factor, to confirm that the F-factor is flanked by terminal repeats (reference sequence: AP015016, LN827561.1). The BamHI restriction sites around the F-plasmid recombined in the YCCEL1 genome and the length of the restriction fragments are indicated in the schematic. One TR repeat is 539 bp in length.

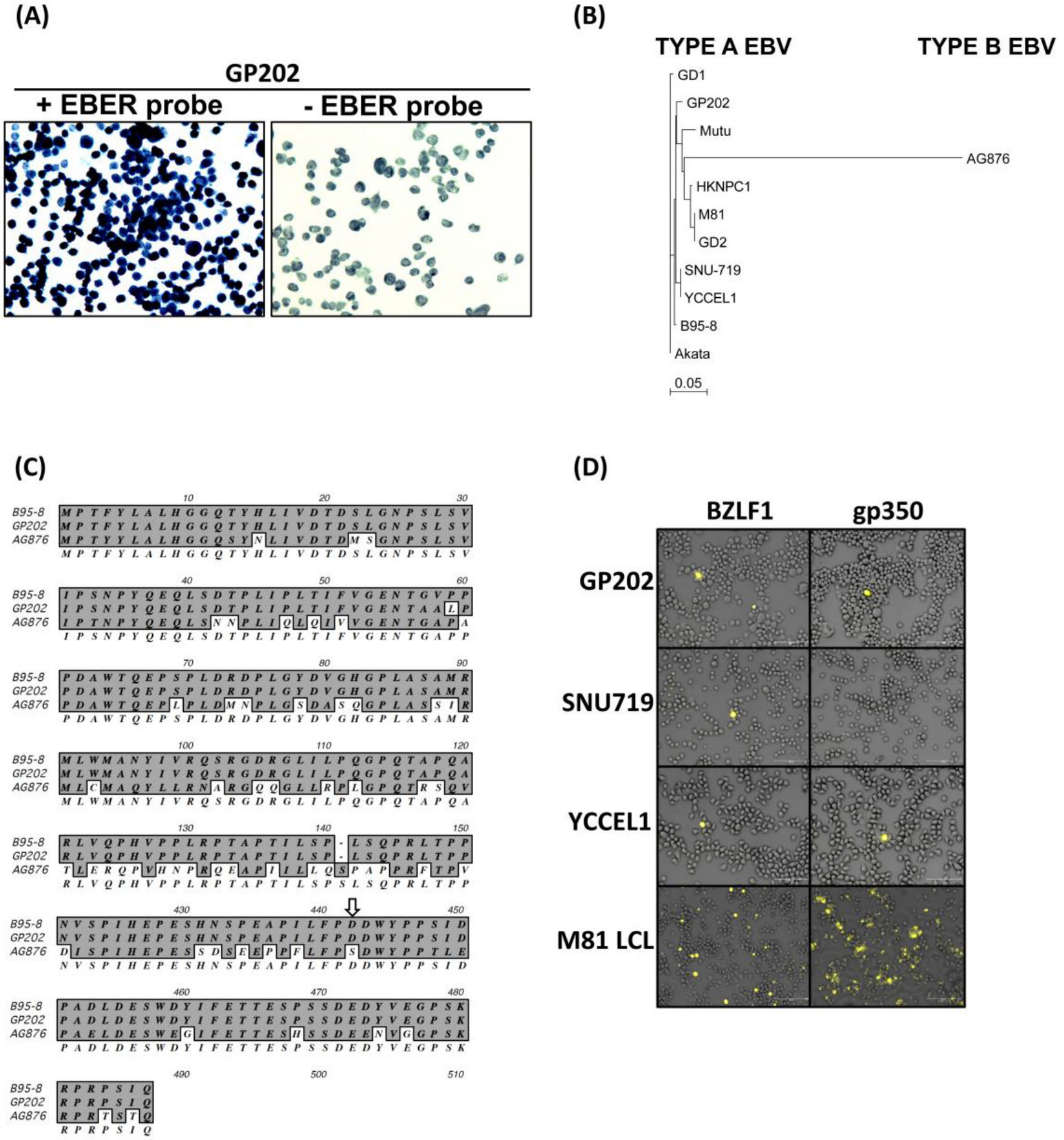

Supplementary Figure 2: GP202, an EBV-positive gastric carcinoma cell line isolated from a gastric carcinoma developed by an European patient. A. We performed an EBER *in situ* hybridization on the GP202 cell line to determine its EBV status. The picture shows the results of the experiment, as well as a control without EBER probe. The cells were counterstained with hematoxylin. **B** and **C.** We sequenced the N- and C-terminal segments of the EBNA2 gene to generate a divergence tree of the EBNA2 protein and show that GP202 is a type A EBV strain. The arrow in (C) points to the polymorphism that defines the type A and B strains. **D.** The gastric carcinoma cell lines YCCEL1, SNU719 and GP202 show evidence of spontaneous lytic replication. The cells were immunostained with antibodies specific to BZLF1 and gp350. M81 LCL was used as a positive control.

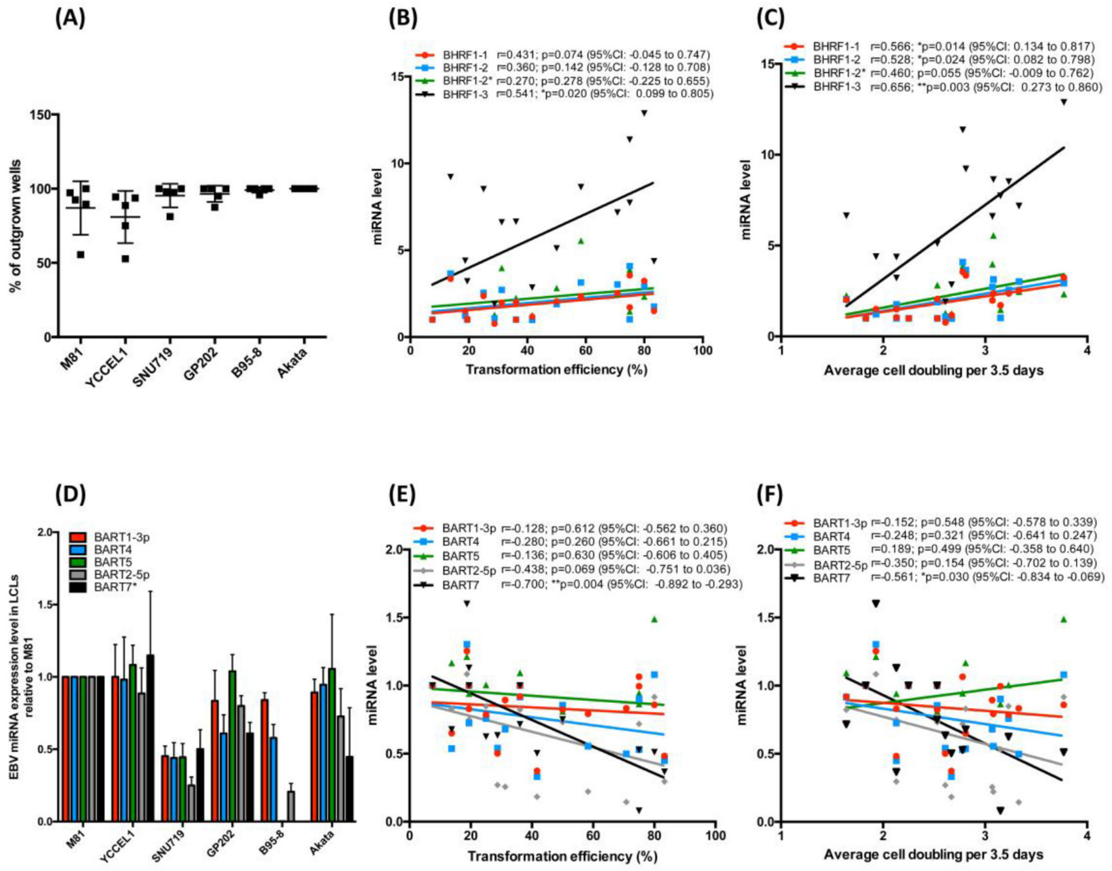

Supplementary Figure 3: Transformation efficiency of B cells infected with different EBV strains and correlation with miRNA expression levels A. This dot plot shows the efficiency of EBV-mediated transformation in infected primary B cell populations seeded at 30 EBNA2-positive cells per well in 96 well cluster plates. The values given represent the percentage of outgrown wells (Also see Figure 1A). **B** and **C.** The figures evaluate the degree of correlation between the expression level of the BHRF1-miRNAs in LCLs and either the transformation efficiency of the different viruses shown in Figure 1A (B) or the cell-doubling rate reported in Figure 1C (C). **D.** The bar graph shows the expression levels of 4 members from the BART miRNAs in the samples shown in Fig. 1C. **E** and **F.** We also show the degree of correlation between the expression level of the tested BART-miRNAs and the transformation ability of the different viruses (E) or the cell proliferation rate in the infected cells (F). The B95-8 BART5 and BART7* miRNAs were excluded from the correlation analyses as B95-8 strain has a deletion in this region. CI: confidence interval. For (B), (C), (E) and (F) we calculated the Pearson *r* correlation coefficients and their accompanying p-values are shown for each condition. 95%CI: 95% Confidence interval.

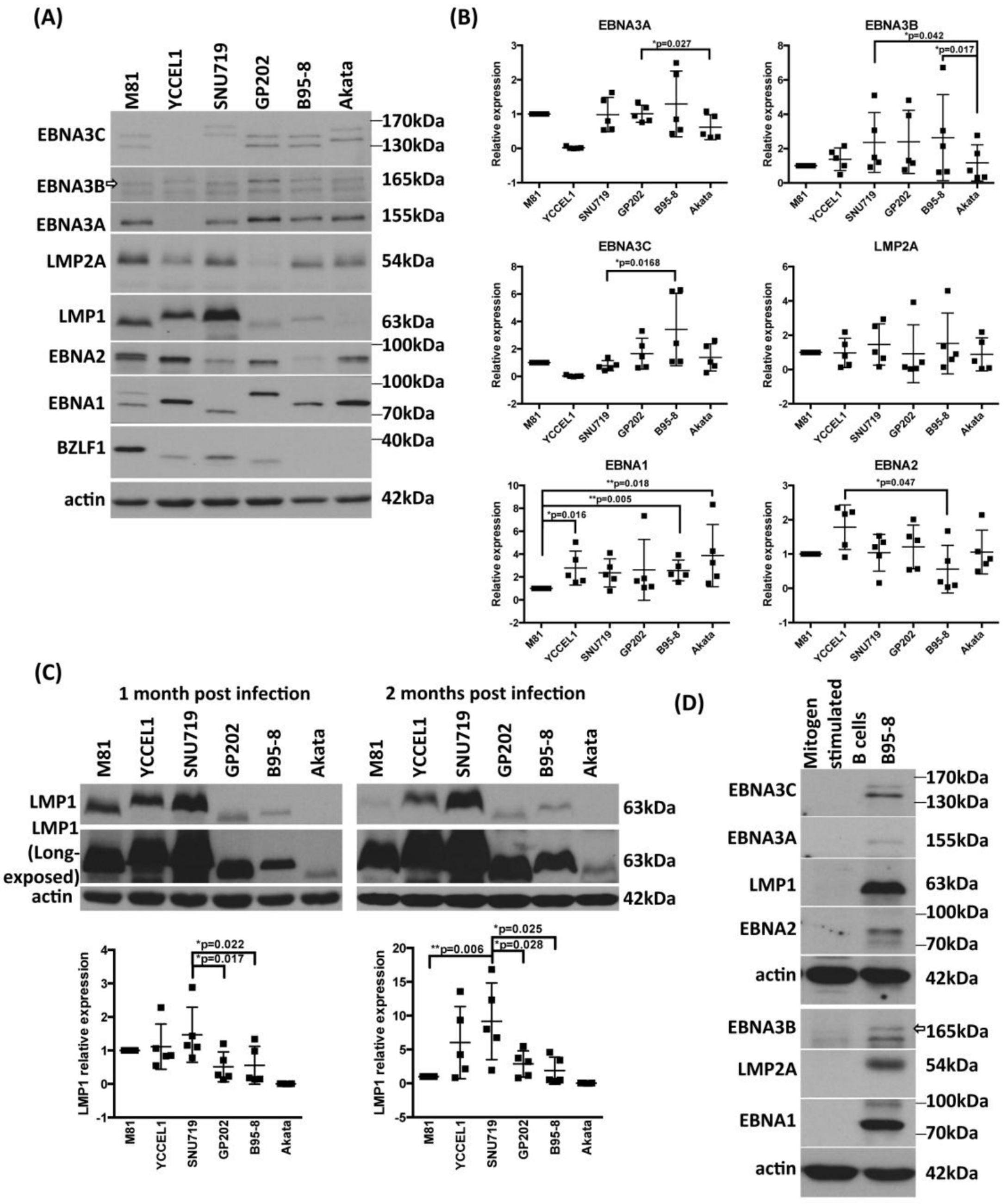

Supplementary Figure 4: EBV latent protein expression pattern in LCLs generated with different EBV strains. We performed immunoblot analyses on LCLs transformed with six different EBV strains using antibodies specific for the EBV latent proteins and an actin-specific antibody used as an internal loading control. **A.** We show the Western blots performed on one blood sample at 1 month post-infection. The arrow indicates the location of the EBNA3B protein. **B.** The 6 dot plots summarize the data obtained from the infection of five individual blood samples with the virus panel 1 month post-infection. All blots were scanned and subjected to signal quantitation using the ImageJ software. The results are given relative to those obtained with M81. The EBNA3A and EBNA3C-specific monoclonal antibodies cannot recognize the YCCEL1 extracts due to polymorphisms of these proteins. **C.** We compared LMP1 expression in established LCLs 1 or 2 months after infection. The figure shows representative Western blots with a LMP1-specific antibody. The graphs show signal intensities recorded after scanning of the blots in 5 independent blood samples. The results are given relative to the values obtained in LCLs infected with M81. **D.** We assessed the specificity of antibodies directed against EBV latent proteins used in this study. We performed Western blots on cell lysates prepared from a LCL transformed by B95-8 and from EBV-negative B cells that had been cultured on CD40-ligand feeder cells supplemented with 25ng/ml IL-4 for 6 days. The antibody specific to actin serves as an internal loading control.

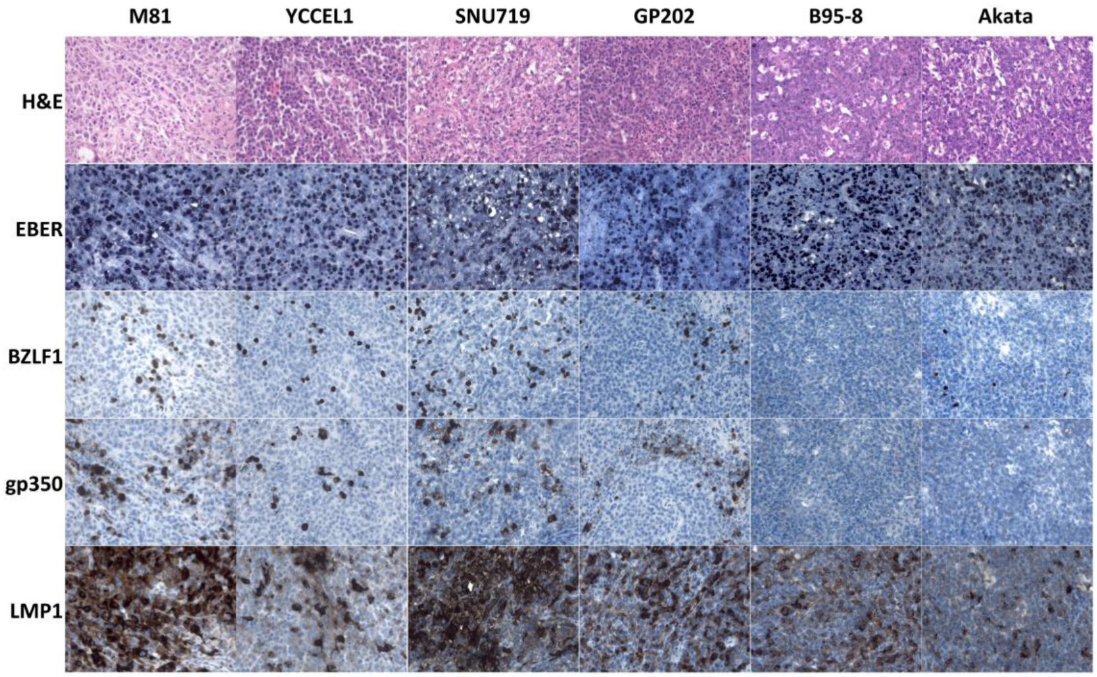

Supplementary Figure 5: Histological analyses of the tumors that developed in NSG mice after injection of B cells exposed to EBV. Consecutive tissue sections of tumor samples developed in gut and pancreas region were stained with hematoxylin and eosin (H&E), immunostained with antibodies specific for BZLF1, gp350, LMP1, or subjected to an *in situ* hybridization with an EBER-specific probe.

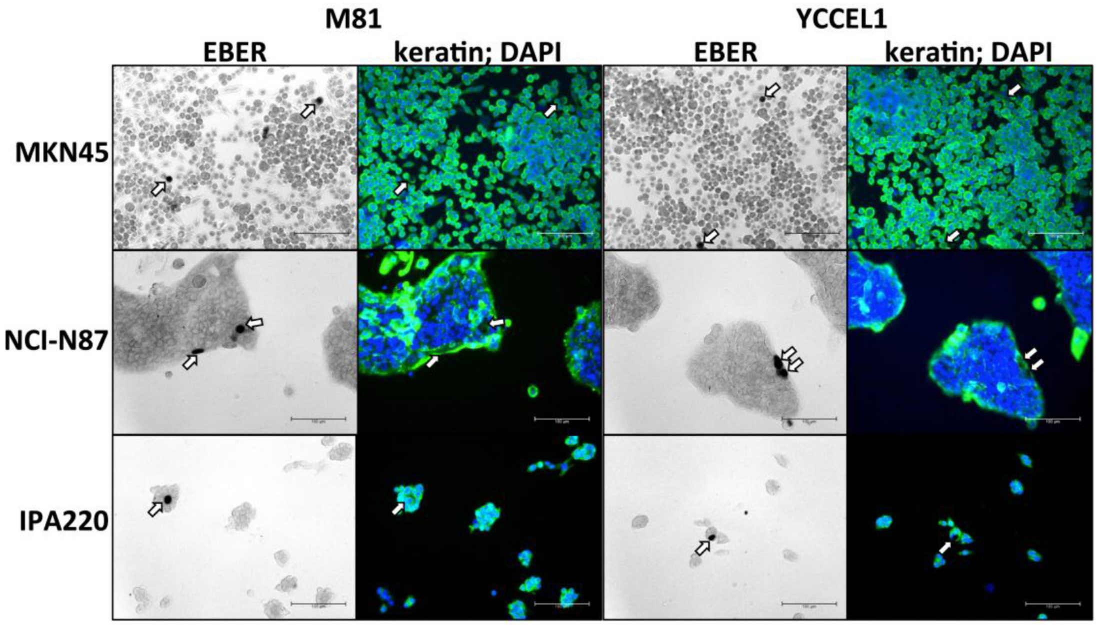

Supplementary Figure 6: Multiple gastric carcinoma cell lines can be infected with some EBV strains, albeit with extremely low efficiency. Three gastric carcinoma cell lines, MKN45, NCI-N87, and IPA220 were infected with M81 and YCCEL1 using B-cell transfer infection. The EBV-infected cells were visualized by *in situ* hybridization with an EBER-specific probe. Arrows indicate the position of EBER-positive cells. The cells were simultaneously stained with an antibody specific to human keratin coupled to FITC (green signals) and the nuclei were counterstained with DAPI (blue signals). This staining confirmed that the EBER-positive cells are truly keratin-positive carcinoma cells and not residual EBV-infected B cells used for transfer infection. (Scale bar: 100 μm).

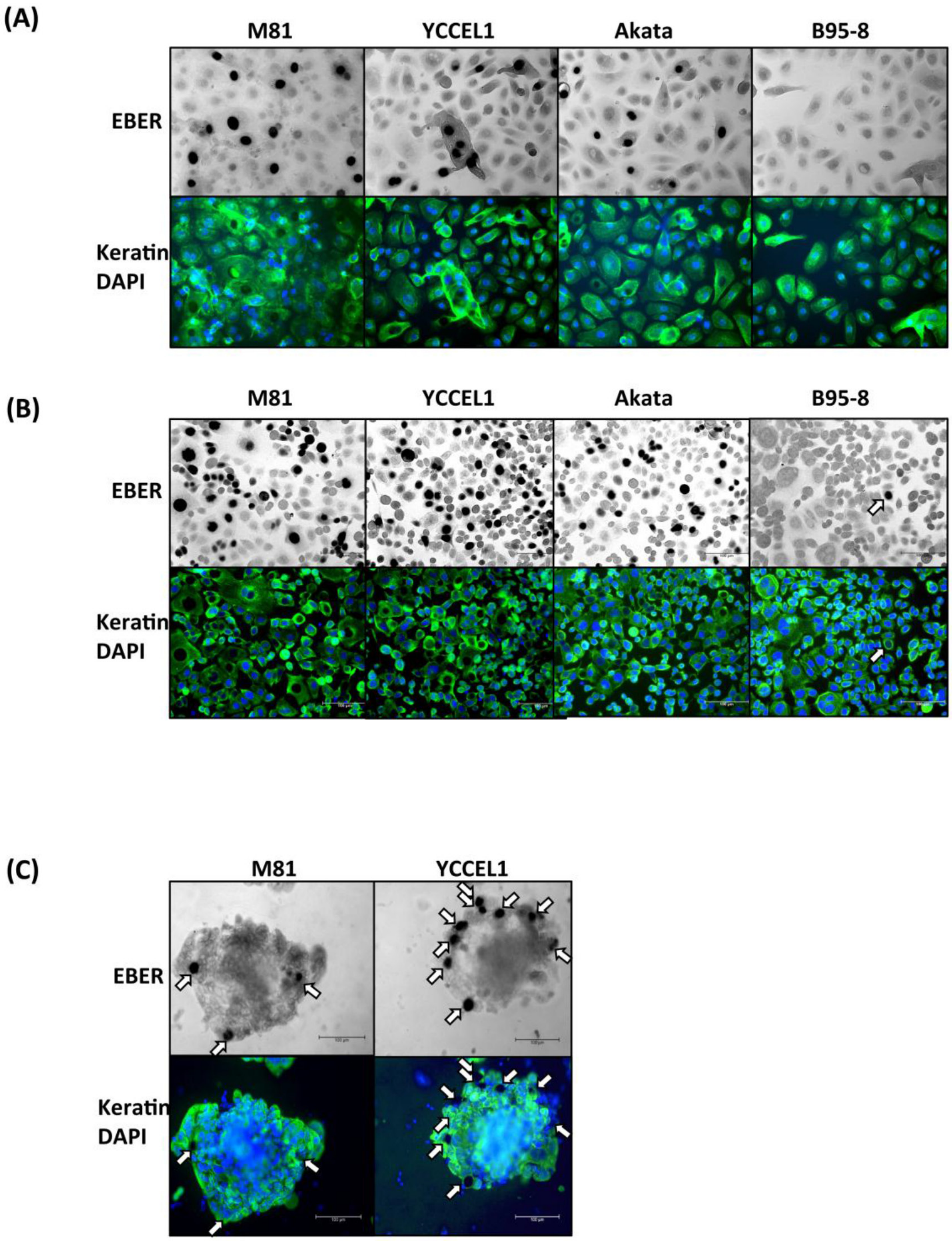

Supplementary Figure 7: EBV transcomplemented with gp110 has an increased infectivity towards epithelial cells. We show representative examples of transfer infections with M81, YCCEL1, Akata or M81 virions complemented with gp110. The infected cells show EBER-positive nuclei. Cells were stained with keratin to confirm their epithelial lineage. **A.** Infection of primary epithelial cells, **B.** Infection of AGS 2D cultures, and **C.** Infection of AGS gastric spheroids. (Scale bar: 100 µm).

Supplementary Table 1: EBV strains included in the study

Strain	Associated Diseases	Country of origin	Virus type [a]
M81	Nasopharyngeal Carcinoma	Hong Kong, China	Recombinant
B95-8	Infectious Mononucleosis	US	Recombinant
Akata	Burkitt's Lymphoma	Japan	Recombinant
YCCEL1	Gastric Carcinoma	Korea	Recombinant
SNU719	Gastric Carcinoma	Korea	Non-recombinant
GP202	Gastric Carcinoma	Portugal	Non-recombinant

[a] The recombinant viruses were produced in HEK293 cells stably transfected with EBV BACMIDs. The non-recombinant viruses were collected from the supernatants of spontaneously replicating marmoset LCLs.

Supplementary Table 2: Macroscopic and histological features of NSG mice treated with EBV-infected primary B cells

virus strain	Mouse number	Time of autopsy (days post infection)	Location of tumors
M81	1	36	Gut/Pancreas, Liver
M81	2	36	Gut/Pancreas
M81	3	42	Gut/Pancreas, Liver
M81	4	42	Gut/Pancreas, Liver
M81	5	42	Spleen, Gut/Pancreas, Liver
M81	6	50	Spleen, Gut/Pancreas, Liver
M81	7	49	Spleen, Gut/Pancreas, Liver
YCCEL1	1	43	Gut/Pancreas, Liver
YCCEL1	2	43	Gut/Pancreas
YCCEL1	3	43	Spleen, Gut/Pancreas, Liver
YCCEL1	4	50	Spleen, Gut/Pancreas, Liver
YCCEL1	5	55	Spleen, Gut/Pancreas, Liver
YCCEL1	6	42	Spleen, Gut/Pancreas, Liver
YCCEL1	7	44	Peritoneal wall
SNU719	1	35	Spleen, Gut/Pancreas, Liver
SNU719	2	35	Spleen, Gut/Pancreas, Liver
SNU719	3	25	Tumor free
SNU719	4	37	Spleen, Gut/Pancreas, Liver
SNU719	5	37	Gut/Pancreas, Liver
SNU719	6	37	Spleen, Gut/Pancreas, Liver
SNU719	7	37	Spleen, Gut/Pancreas, Liver
GP202	1	35	Spleen, Gut/Pancreas, Liver
GP202	2	35	Spleen, Gut/Pancreas, Liver
GP202	3	35	Spleen, Gut/Pancreas, Liver
GP202	4	35	Gut/Pancreas
GP202	5	37	Spleen, Gut/Pancreas, Liver
GP202	6	37	Spleen, Gut/Pancreas, Liver
GP202	7	37	Spleen, Gut/Pancreas, Liver
B95-8	1	30	Tumor free
B95-8	2	36	Gut/Pancreas, Liver
B95-8	3	30	Gut/Pancreas
B95-8	4	56	Gut/Pancreas
B95-8	5	37	Gut/Pancreas, Liver
B95-8	6	37	Gut/Pancreas, Liver
B95-8	7	56	Tumor free
Akata	1	36	Gut/Pancreas, Liver
Akata	2	36	Spleen, Gut/Pancreas, Liver
Akata	3	36	Gut/Pancreas, Liver
Akata	4	30	Gut/Pancreas
Akata	5	47	Spleen, Gut/Pancreas
Akata	6	56	Tumor free
Akata	7	30	Spleen, Gut/Pancreas
